# Book Reviews

**Published:** 1897-05

**Authors:** 


### *BOOK REVIEWS.*

Lectures on Renal and Urinary Diseases. By Robert Saundby, M.D., Edin.; Fellow of the Royal College of Physicians, London, etc. With numerous illustrations. Second edition. Philadelphia, W. B. Saunders, 925 Walnut street. 1897. Price, $2.50.

The complete and exhaustive manner in which the author has treated his subject commends itself to careful consideration, and his reputation and vast clinical experience give additional weight of worth.

A division into four sections is made of the book, which rather adds to its attractiveness.

Section 1 is devoted to a discussion of Bright's disease; and while this general English term for renal inflammation has been discarded by American writers for more technical appellations, the words of the writer lose none of their force by this nomenclature.

The anatomy of the kidney is reviewed, and judicious illustrations accompany the text.

Concerning the "Pathology of Albuminuria," Dr. Saundby lays great stress upon the import of this condition, and adopts the advanced idea that the presence of albumen in the urine is not *per se* an infallible sign of serious pathologic state, nor a menace to man's normal tenure of life.

In this section much space is given to the pathology of dropsy, polyuria, and uremia; to the pathological relation of tube casts; to the various organic and functional changes occurring in the course of the disease; to the various complications, and, finally, to the treatment.

Section 2 is a study of the urine, with many well written pages upon its clinical examination.

Section 3 treats of diabetes, mellitus and insipidus, giving the most modern views regarding the etiology, morbid anatomy and treatment of these conditions.

Section 4 discusses stone in the kidney, hydronephrosis, pyonephrosis, pyelitis, hematuria, and hemoglobinuria.

The work is a modern exposition of the views held upon these subjects, and is thoroughly in accord with the most advanced ideas regarding their pathology and treatment. An extensive bibliography accompanies each section. M. A. P.

---

THE DISEASES OF THE STOMACH. By Dr. C. A. Ewald, Extraordinary Professor of Medicine at the University of Berlin; Director of the Augusta Hospital, etc. Translated and edited, with numerous additions, from the third German edition, by Morris Manges, A.M., M.D., Assisting Visiting Physician to Mt. Sinai Hospital. Second revised edition. Published by D. Appleton & Co., New York.

"The great progress which has been made in our knowledge of the diseases of the stomach since the appearance of the first edition of this work, in 1892, has rendered a new edition necessary.

"I would direct attention to the progress made during the past few years in gastric surgery, which has now passed beyond the stage of a few isolated, daring operations. In the discussion of the various diseases, I have considered the indications for operative interference, and have presented the pros and cons so far as would be necessary to enable a physician to determine whether in a concrete case the aid of the surgeon might be required.

"The present book, based upon lectures which were delivered before practitioners, and which were subsequently enlarged, has been designed for general practitioners and students; every part has been considered from this standpoint, and represents an extensive practical experience."—*From the Preface.*

In giving the medical profession this second revised translation of Professor Ewald's treatise on the diseases of the stomach, Dr. Manges has placed the profession under even greater obligations than we owed for the first. The first translation was then an almost exhaustive treatise, and now, with so much new and valuable data added, the work is a *sine qua non.*

The only criticism I can make is, that the cuts and illustrations might be better; but this slight defect does not militate against the great merit of the author's effort and translator's fidelity.

The book is printed in good type and is well bound, and can be procured through the Southern branch of D. Appleton & Co., Gould Building, Atlanta, Ga. H. H.

---

POCKET MEDICAL DICTIONARY. Comprising Pronunciation and Definition of 10,000 Essential Words and Terms Used in Medicine and Associated Sciences. By William R. Warner. Published by William R. Warner & Co., Philadelphia.

"In compiling this dictionary of medicine the publishers have endeavored to render a concise yet perfectly comprehensive pronunciation and definition of each word." Pocket dictionaries are apt to be too concise to be of much service, and definitions are often almost worthless. This one is good proof of this statement. *Groin* is defined as "angular curve above thigh"; the *latissimus dorsi* muscle as a "flat sheet of muscle springing from dorsal vertebræ"; *gladiolus* is "sternal center"; *grain* is "$\frac{1}{20}$℈"; the *elbow* is "joint uniting radius and humerus"; etc. A first-course student might find this little work of some assistance, but we think its deficiencies would soon become realized and he would have to seek a larger work for his real information.

